# Droplet size prediction in the production of drug delivery microsystems by ultrasonic atomization

**Published:** 2013-09-02

**Authors:** Annalisa Dalmoro, Matteo d’Amore, Anna Angela Barba

**Affiliations:** 1 Dipartimento di Farmacia, Università di Salerno, Salerno, Italy; 2 Dipartimento di Ingegneria Industriale, Università di Salerno, Salerno, Italy

**Keywords:** Ultrasonic atomization, dimensionless numbers in atomization, microparticles size prediction

## Abstract

Microencapsulation processes of drugs or other functional molecules are of great interest in pharmaceutical production fields. Ultrasonic assisted atomization is a new technique to produce microencapsulated systems by mechanical approach. It seems to offer several advantages (low level of mechanical stress in materials, reduced energy request, reduced apparatuses size) with respect to more conventional techniques. In this paper the groundwork of atomization is briefly introduced and correlations to predict droplet size starting from process parameters and material properties are presented.

## I. INTRODUCTION

Microencapsulation is used to modify or to delay drug release. It offers greater effectiveness, lower toxicity and more lasting stability than conventional formulations. Spray-drying technique for preparation of microsystems presents several advantages compared to other microencapsulation techniques: it is, in principle, a continuous process, giving good reproducibility and potential for scale-up. Spray is usually generated by pressure and rotary nozzles, but they have some disadvantages, such as lack of control over the mean droplet size, broad droplet distributions, risk of clogging in case of suspensions. Moreover, they use only a small amount of their operating energy (centrifugal, pressure or kinetic energy) to shatter the liquid, while most of this energy is transformed into kinetic energy of the particles thus large settling chambers are required (summarizing: costs increase when speed of the atomized particles; increases) [ 1 ] . These disadvantages can be reduced using an ultrasonic atomizer: the ultrasound energy is transmitted with high efficiency to the liquid by a sonotrode, causing atomization. Despite ultrasonic nozzles have not been routinely used in laboratory scale spray-drying equipment, they can offer the generation of droplets, and consequently of microparticles, with a uniform size distribution [ 2 ] .

In general, the atomization is defined as the disintegration of a liquid in drops in a surrounding gas by an atomizer [ 3 ] . The resultant suspension is defined as spray, mist, or aerosol. Atomization occurs owing to the competition between destructive and cohesive forces on the liquid surface, leading to fluctuations and disturbances in the liquid. The cohesive effect of liquid surface tension keeps the fluid in a status showing the lower surface energy, and the stabilizing effect of the viscosity tends to oppose any variation in liquid geometry. Instead, the external forces, such as aerodynamic, centrifugal, and electrostatic forces, act on the liquid surface promoting its disintegration. The initial process of disintegration or break-up is defined as primary atomization. However a number of larger droplets produced in the primary atomization can be unstable, thus reducing into smaller droplets. This process is usually defined as secondary atomization.

The effect of the forces acting on the liquid is resumed by the dimensionless numbers:

(1) Re=ρ⋅u⋅dpμ

(2) $$\mathrm{We}=\frac{\rho \cdot u^{2}\cdot d_{p}}{\sigma }$$

(3) Oh=WeRe=μρ⋅σ⋅dp

Where:

*
Re
*: Reynolds number;
*
We
*: Weber number;
*
Oh
*: Ohnesorge number;
*
ρ
*: liquid density;
*
u
*: liquid velocity;
*
d _p
_*: jet diameter (primary atomization) or drop diameter (secondary atomization);
*
μ
*: liquid viscosity;
*
σ
*: surface tension.

The Reynolds number expresses the ratio between inertial and viscous forces. The Weber number is a measure of the relative importance of the fluid’s inertia compared to its surface tension. By combining the two dimensionless numbers to eliminate the liquid velocity, the Ohnesorge number, containing fluid properties, is obtained. Thus, droplets diameter can be predicted by correlations mainly based on liquid properties (density, viscosity, surface tension), on atomizer geometry (orifice size) and on operative parameters, such as liquid flow rate. However, physical phenomena involved in the atomization processes have not been understood yet to such an extent to allow droplet size to be expressed by equations directly derived from the first principles. The correlations proposed are mainly based on empirical studies, even if the empirical correlations have been proved to be a practical way to determine droplet sizes from process parameters and relevant liquid/gas physical properties [ 3 ] . The empirical correlations are very useful in the forecast of microparticles size, especially if this feature plays a key role. In pharmaceutical applications microparticles size can affect rate and duration of release of entrapped therapeutic agents.

## II. ULTRASONIC ATOMIZATION

When a liquid flows on a vibrating surface and splits into fine droplets, ultrasonic atomization occurs. A correlation proposed for the size diameter prediction of droplets produced by ultrasonic atomization, mainly based on the frequency *f* , was given by Lang [ 4 ] :

(4) $$d_{p}=0.34\cdot {\left(\frac{8\cdot \pi \cdot \sigma }{\rho \cdot f^{2}}\right)}^{1/3}$$

This correlation is only applicable when liquid phase viscosity and liquid flow rate have no effect on droplet size, but these parameters were proven to be very important in ultrasonic atomization. The dimensionless numbers, which dictate the droplets size, were modified in order to consider the dependence on physical-chemical properties and ultrasonic parameters. Therefore, the concept of critical Weber, for which inertial and surface tension forces are equilibrated ( *We* _c_ = 1) was extended to ultrasonic atomization, indicating the critical flow rate, *Q* _c_ , as the threshold above which the flow rate influences the droplets size [ 5 ] . The critical flow rate was defined as:

(5) $$Q_{c}=\frac{\sigma }{f\cdot \sigma }$$

Then, the maximum flow rate, above which dripping takes place forming larger droplets, is considered. The maximum flow rate is the volumetric displacement rate of vibrating surface, given by the product of frequency, *f* , amplitude of sound wave, *Am* , and area of vibrating surface, A. The amplitude, *Am* , is defined as:

(6) $$\mathrm{Am}=\frac{1}{2\pi \cdot f}\cdot \sqrt{\frac{2I}{\rho \cdot C}}$$

where *I* is the power surface intensity (defined as the ratio between the power delivered at the surface, *P* , and the area of vibrating surface, *A* ) and *C* is the speed of sound. The Weber number was thus modified to include the flow rate, *Q* , and the ultrasonic frequency, *f* :

(7) $$\mathrm{We}=\frac{f\cdot Q\cdot \rho }{\sigma }$$

The Ohnesorge number was also modified taking into account that in ultrasonic atomization the growth of instability is given by the amplitude, *Am* :

(8) $$\mathrm{Oh}=\frac{\mu }{f\cdot Am^{2}\cdot \rho }$$

Another dimensionless number, called Intensity number, I _N_ , is defined to take into account the effect of energy density on droplets size:

(9) $$I_{N}=\frac{f^{2}\cdot Am^{4}}{C\cdot Q}$$

From these dimensionless numbers, an universal correlation was proposed by Rajan and Pandit [ 5 ] :

(10) $$\begin{array}{c} d_{p}={\left(\frac{\pi \sigma }{\rho \cdot f^{2}}\right)}^{0.33}[1+A\cdot {\left(\mathrm{We}\right)}^{0.22}\cdot {\left(\mathrm{Oh}\right)}^{0.166}\\ \cdot {\left(I_{N}\right)}^{-0.0277}] \end{array}$$

The exponents in this correlation were chosen from experimental observations reported in literature.

Ramisetty et al. [ 6 ] also carried out experiments and developed a correlation applicable in the following ranges: *f* = 20–130 KHz; ρ = 912–1151 Kg m ^−3^ ; σ = 0.0029–0.073 N m ^−1^ ; Oh = 2.71 – 161.64; We = 14.8 – 571; I _N_ = 3.65*10 ^−13^ – 1.92*10 ^−9^ . The correlation was:

(11) $$\begin{array}{c} d_{p}=0.00154\cdot {\left(\frac{\pi \sigma }{\rho \cdot f^{2}}\right)}^{0.33}[1+{\left(\frac{\pi \sigma }{\rho \cdot f^{2}}\right)}^{-0.2}\cdot {\left(\mathrm{We}\right)}^{0.154}\\ \cdot {\left(\mathrm{Oh}\right)}^{-0.111}\cdot {\left(I_{N}\right)}^{-0.033}] \end{array}$$

Avvaru et al. [ 7 ] made an attempt to include the rheological nature, pseudo-plasticity (non-Newtonian behaviour) of the atomizing liquid. In particular, they collected data to obtain a correlation for an aqueous solution of carboxy methyl cellulose (CMC), having a shear thinning behavior, with a flow behavior index *n* :

(12) $$\begin{array}{c} d_{p}={\left(\frac{\pi \sigma }{\rho \cdot f^{2}}\right)}^{0.33}+0.0013\cdot {\left(\mathrm{We}\right)}^{0.008}\cdot {\left(\mathrm{Oh}\right)}^{-0.14/n}\\ \cdot {\left(I_{N}\right)}^{0.28} \end{array}$$

Barba et al. [ 8 ] proposed a modification of the correlation (10) by applying it to the ultrasonic atomization of alginate solutions:

(13) $$\begin{array}{c} d_{p}=0.058\cdot {\left(\frac{\pi \sigma }{\rho \cdot f^{2}}\right)}^{0.33}\cdot {\left(\mathrm{We}\right)}^{0.151}\cdot {\left(\mathrm{Oh}\right)}^{0.192}\\ \cdot {\left(I_{N}\right)}^{-0.02} \end{array}$$

Therefore, for the atomization of both Newtonian and non-Newtonian liquid, the following observations were done.

The droplet size decreases by increasing the frequency, *f* . At higher *f* , the liquid is subjected to a larger number of compression phases, thus the crest growth is reduced causing the eventual decrease of droplets size.

There is a range of liquid flow rate influencing the droplets size. Below a critical flow rate, *Q* _c_ , the liquid cannot cover the whole atomizing surface, thus no effective atomization occurs. Above *Q* _c_ , size is proportional to the liquid flow rate, again basing on film thickness on the atomization surface. Above a maximum flow rate, dripping occurs.

An increase in ultrasonic power causes an increase of the vibration amplitude ( *Am* ), leading to a broader distribution of droplets size. In effect, when power delivered to the tip is low, it can be freely used as soon as liquid spreads on the atomizer surface. Instead, at higher power, the liquid delivered on the surface immediately atomizes causing both a conical pattern of the spray and the exposition of the external part of the atomizer to air, being not wetted by the liquid.

The influence of viscosity becomes significant when it is greater than 10 cP [ 5 ] . The increase of liquid viscosity was shown to cause a reduction of droplets size. As the liquid viscosity increases, the liquid cannot be immediately atomized as it comes out from the hole. Therefore, the residence time of the liquid on the atomizing surface increases, causing liquid temperature rising, owing to the vibrational energy dissipation, and consequent decrease of liquid viscosity to a critical value. Thus, the liquid atomizes like a low viscosity liquid giving a lower droplets size. The decrease of liquid viscosity is also enhanced by the shear thinning behavior of liquids, such as CMC and alginate: the apparent viscosity on the vibrating surface decreases to the high shear rates. As a result, a pseudo-plastic (non-Newtonian-shear thinning) liquid has a lower droplets size than a viscous Newtonian liquid, with a viscosity equal to the zero shear rate viscosity of the shear thinning liquid.

When surface tension decreases, at higher amplitudes the number of capillary waves per unit of vibrating area increases, causing the immediate ejection of droplets from the crests. The higher number of droplets ejected from a liquid film for a same liquid flow rate causes a corresponding decrease in droplets size.

Overall, a proper control of the equipment operating parameters (flow rate, frequency, power) and liquid properties (liquid type, viscosity and surface tension) can give the desired droplets size.

In this study loaded microparticles of a natural polymer which have an interest as pharmaceutical dosage forms [ 9 ] , were achieved starting from ultrasonic atomization and drying processes. Fine droplets sizes were measured and calculated using literature correlations.

## III. METHODOLOGY

A biocompatible polymer, alginate with high guluronic content ( *Manugel GHB Sodium alginate, FMC Bio-polymer* ), giving more rigid gels, that are less subjected to swelling and erosion [ 10 ] , was used as drug carrier for the encapsulation of a lipophilic model molecule, α-tocopherol vitamin ( *Sigma Aldrich* ). The ultrasonic device used to produce micro-particles ( *Sonics & Materials Inc., CT, USA; broadband ultrasonic generator mod. 06- 05108- 25 KHz Sono-Tek corporation, NY, USA* ) was provided of a dual liquid feed probe ( *Sono-Tek 025-00010, Sono-Tek corporation NY, USA* ). The process of particles production was already described in a previous work [ 11 ] . Briefly, the internal ( *core* ) and the external ( *shell* ) solutions (both having concentrations of alginate 1.5% w/w) were fed to the coaxial atomizer by peristaltic pumps ( *Verderflex OEM mod. Au EZ, RS Components Milan IT* , controlled by two systems with variable tension *Long WEI DC power supply PS305D* to adjust the delivered solutions flow rate). In particular, the core solution was made of alginate 1.5% (w/w), α-tocopherol 1% (w/w) and Tween 80 0.5% (w/w). The surfactant Tween 80 was introduced in the core solution to reduce interfacial tension, and consequently droplet dimensions; moreover a concentration of 0.5% (w/w) was needed to obtain a stable emulsion of the water insoluble α-tocopherol with the water soluble solution of alginate. The two solutions came in contact only at the exit of the two channels, at the atomizer’s tip, where they were nebulized, using a power of 10 W, chosen to ensure an uniform distribution of the spray. The atomization products (micro-drops) were collected in a cross-linking solution at a concentration of 8.9 g/l of CaCl _2_ , submitted to agitation for 5 min in a beaker. All the parameters defined for the apparatus exercise are reported in Table 1 .

By crosslinking process, fine particles with matrix (only core) and shell-core structures were obtained. They were then separated by filtration, washed, photographed (by *Canon digital camera, IXUS 850 IS, by Leica digital camera DFC 280* mounted on an optical microscope, *Leica Microsystems DM-LP, Wetzlar, D* ) and subjected to diameter measurements by image analysis (using the public domain *software ImageJ 1.40g, Wayne Rasband, National Institutes of Health, USA,* freely available at http://rsb.info.nih.gov/ij/).

Finally the produced microdroplets were dried by a microwave oven ( *De Longhi mod. Easy* ) until a constant weight was reached.

## IV. RESULTS AND DISCUSSION

**Fig. 1:**
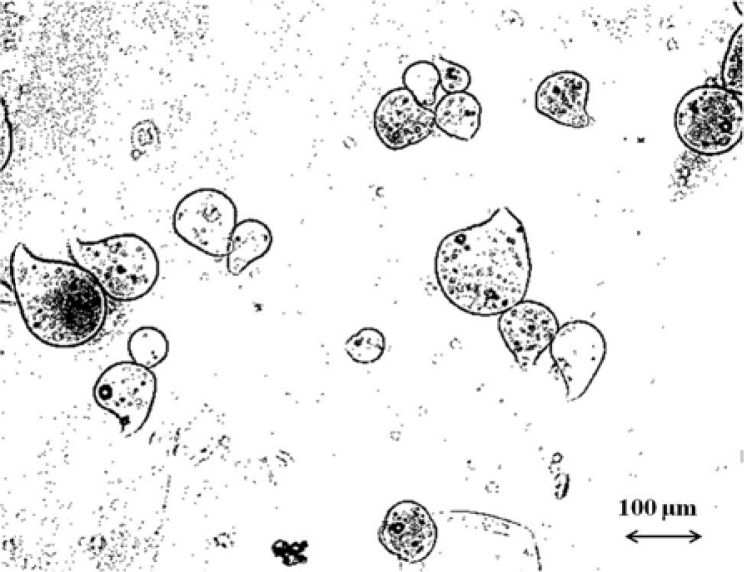
Optical microscope pictures of fresh shell-core microdroplets.

Produced microdroplets ( Fig. 1 ) were characterized in terms of morphology, size, and drug loading/release properties.

The used spray conditions (both internal and external solution concentrations and feed rates, atomizer’s frequency and power) were selected to obtain microsystems able to encapsulate the active molecule. In effect, the microsystem, as discussed in a previous work [ 11 ] , showed peculiarities (high loading, delayed release) that make them of interest for specific drug delivery applications.

**Fig. 2:**
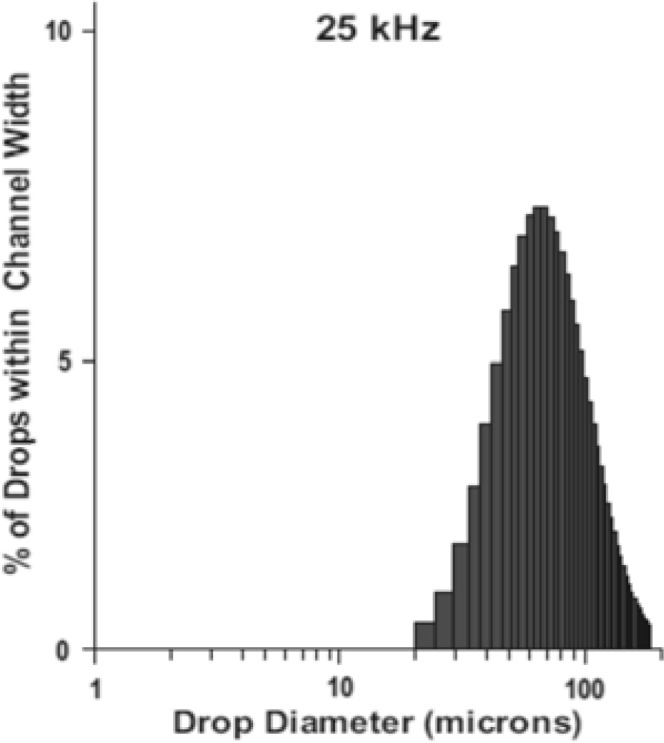
Drop diameter distribution for 25 KHz ultrasonic atomizer (data for water)

Image analysis for size control of both shell-core and matrix micro-particles, done on both fresh and dried product, showed a narrow size distribution ( Table 2 ). Shell-core and matrix droplets (or fresh microparticles) have an initial size of 78 μm and 76 μm respectively. This values are in good agreement with data (related to water) of fine drop diameter distribution given by Sonotek ( Fig. 2 ). For water atomized by 25 kHz nozzle distribution, 0.7% of all drops falls in the channel that covers the 10–15 m range, 1.5% falls in the following 15–20 m range, and so on. The higher percentage of particles falls in a dimensional range between 70 and 80 m, as well as for alginate particles.

After drying the particle size is reduced, by volumetric shrinkage of about 85%, to about 40 μm, as highlighted in Table 2 . Moreover, fresh droplets assume a light pendant shape, as showed in Fig. 1 , because a spherical drop deforms due to the impact then keeps the shape owing to the fast cross-linking [ 8 ] . This shape is essentially preserved in dried particles.

Literature correlations, described in the paragraph “ULTRASONIC ATOMIZATION”, were applied to predict the size of microsystems produced in this apparatus. The physical properties of alginate solution at 1.5% (w/w) ( Table 3 ) were taken by Chan et al. [ 12 ] .

The alginate solutions have a non-Newtonian behavior; therefore the solution viscosity can be described by the power law:

(14) $$K\cdot {\dot{\gamma }}^{n-1}$$

The consistency index, *K* , and the flow index, *n* , are functions of the alginate concentration, *c* . Literature fitting equations describe this relationship in a range of alginate concentration between 1% and 3% [ 8 ] :

(15) $$K(c)=0.0619\cdot c^{2.953}$$

(16) n(c)=0.9635⋅exp(-0.08⋅c)

Where *c* is in %, *K* is in Pa·s ^n^ , *n* is dimensionless.

The shear rate at the wall, for a power-law fluid having a volumetric flow rate *Q* flowing in a circular tube of radius *R* is given by the following equation [8] :

(17) $${\dot{\gamma }}_{W}={\left[\left(\frac{1}{n}+3\right)\cdot \frac{Q}{\pi \cdot R^{3}}\right]}^{n}$$

For the used alginate solution, at a concentration of 1.5%, the consistency index, *K* , and the flow index, *n* , were 0.205 Pa·s ^n^ and 0.854, respectively. The calculated shear rate at the wall, from equation (17) , was 333 1/s; the consequent viscosity (from equation 14 ) was 0.088 Pa·s. It is important to highlight some considerations:

- shell-core systems were considered homogeneous owing to the same properties of the material used for both shell and core channels and to the predominance of the shell-channel flow rate;
- actual surface tension is lower than the value reported in literature owing to the presence of the surfactant Tween 80.

**Fig. 3:**
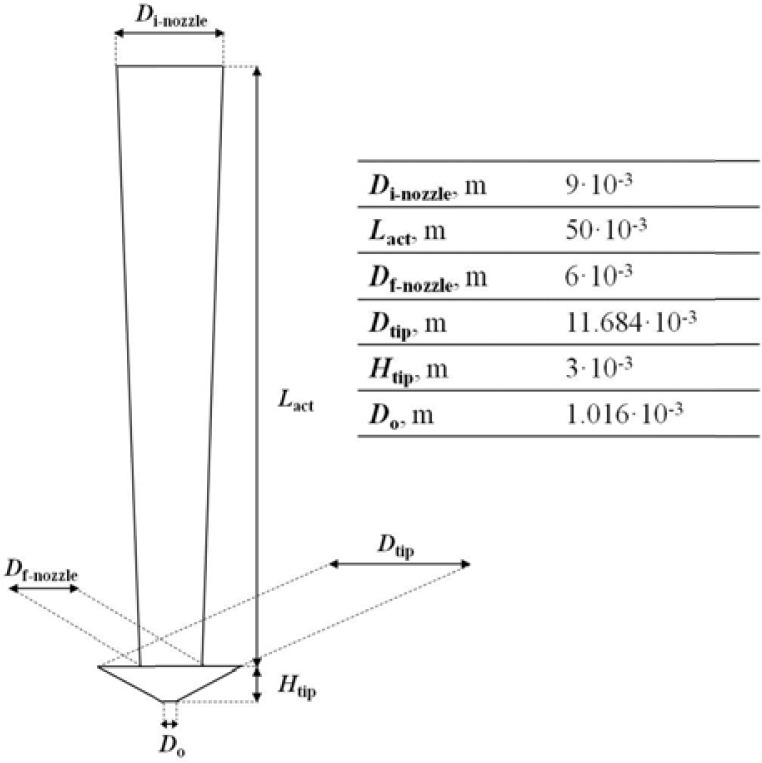
Nozzle geometry and relevant dimensions

Liquid viscosity, atomizer geometry and liquid flow rate have effect on the droplet size in ultrasonic atomization, as previously discussed. In effect, in the calculation of modified dimensionless numbers for ultrasonic atomization, a number of parameters related to the nozzle configuration have to be considered. Nozzle geometry and relevant dimensions are shown in Fig. 3 ; other parameters and combination of some of them are in Table 4 .

Dimensionless numbers assume thus the following values:

- from equation (3.10): *We* = 25.1;
- from equation (3.11): *Oh* = 8.96·10 ^3^ ;
- from equation (3.12): IN = 9.14·10 ^−13^ .

The Weber number is larger than *We* _c_ (=1), thus spray formation is assured. Moreover, the Ohnesorge number is very high owing to the high value of solution viscosity (0.13 Pa·s against 0.001 Pa·s of water).

Finally, both critical and maximum flow rate were evaluated to compare them with experimental flow rates, that, as showed in Table 1 , are 1.1 mL·min ^−1^ (1.83·10 ^−8^ m ^3^ ·s ^−1^ ) and 4.2 mL·min ^−1^ (7·10 ^−8^ m ^3^ ·s ^−1^ ), respectively for core and shell channels. The calculated critical flow rate was *Q* _c_ = 2.8·10 ^−9^ m ^3^ ·s ^−1^ and *Q* _max_ = 2.2·10 ^−5^ m ^3^ ·s ^−1^ , thus experimental flow rates are well collocated within the range of good atomization. Therefore, the three correlations previously described, were applied for droplets size prediction. It is worth to note that both shell-core and matrix microdroplets showed the same size, independently from the flow rate. This phenomenon can be explained owing to the high viscosity of alginate solutions (non-Newtonian shear thinning) that avoids the immediate the detachment of droplet from the capillary waves formed on the surface, determining a similar droplets size distribution in a relatively small range of flow rates (in this case 1.1 and 4.2 mL·min ^−1^ ).

Also, alginate microdroplets obtained by using a higher concentration of the stabilizer Tween 80, introducing it in both shell and core material (compared to the described procedure, where Tween 80 is only in the core, thus less influencing) had a size of 54 m, lower than the size of 76–78 μm. This difference in size is due to the presence of Tween 80, which acts in two ways in the reduction of droplets diameter. First, it reduces the surface tension. Then, the presence of stabilizer gives a higher liquid viscosity, which causes an increase of duration of liquid contact with atomizing surface. The larger time of contact provokes an increase in liquid temperature and the consequent viscosity reduction. Therefore, the combined effect of reduced surface tension and reduced viscosity leads to the reduction in droplets size. However, having alginate/α-tocopherol a lower concentration of Tween 80, properties values in absence of Tween 80 (pure alginate) can be used in the correlations.

The first correlation (11) proposed by Ramisetty et al. [ 6 ] , giving a theoretical drop diameter of 58 m, was not able to predict drop size because the Ohnesorge number is beyond the limits of validity y of this correlation ( *Oh* = 1.32·10 ^4^ >> 2.71–161.64). In effect the alginate solution has a shear-thinning behavior, that is instead taken into account in the correlation (12) proposed by y Avvaru et al. [ 7 ] . The drop diameter predicted by the correlation (12) was of about 71 m, nearer to the measured drop size.

Furthermore, the third correlation (13), proposed by Barba et al. [ 13 ] , gives a predicted particle size of 76 m, which corresponds to the value experimentally obtained, confirming the goodness of this correlation (13) tuned on alginate rather than on CMC, as the previous one (12), that is in any case a good interpretation of the phenomena occurring.

## V. CONCLUSIONS

In this study the groundwork of atomization was briefly introduced; thus the attention was focused on the ultrasonic atomization. In particular, three empirical correlations, which take in account feed solution properties / process parameters, to predict droplets size, were applied. Two correlations have been found reliable to predict droplets size of alginate solution, an interesting biomaterial to produce drug delivery microsystems.

## Figures and Tables

**Table 1:** parameter selected for particles production

** Parameters **	*** Core ***	*** Shell ***
Flow rate, ml/min	1.1	4.2
Alginate, % w/w	1.5	1.5
a-tocopherol, % w/w	1	-
Tween 80, % w/w	0.5	-

Atomization time, min	2	
Cross-linking time, min	5	

**Table 2:** mean size and standard deviation for both shell-core and matrix microparticles, fresh and dried; volumetric shrinkage percentage for the dried ones

	** Mean size, μm **	** S. D. **	** Shrinkage, % **
Shell-core microparticles FRESH	78	±20	
Matrix microparticles FRESH	76	±20	
Shell-core microparticles DRIED	41	±12	86
Matrix microparticles DRIED	40	±12	85

**Table 3:** physical properties of manugel ghb alginate solutions [ 12 ]: the properties of a solution with a concentration of 1.5% ( w / w ) are highlighted

Alginate concentration, % w/w	Density, Kg·m ^ −3 ^	Viscosity, Pa·s	Surface tension, N·m ^ −1 ^
0.5	999	0.038	0.071
** 1.5 **	** 1004 **	** 0.13 **	** 0.07 **
2.5	1008	0.56	0.069
4.0	1017	2.7	0.057
5.0	1023	4.7	0.047

**Table 4:** values to insert in correlations for droplet size prediction in ultrasonic atomization

Area of vibrating surface, * A * , m ^ 2 (*)^	1.38·10 ^ −3 ^
Frequency, * f * , Hz	25000
Delivered power, * P * , W	10
Power intensity, * I * , W·m ^ −2 (**)^	7.25·10 ^ 3 ^
Speed of the sound (in the water), C, m·s ^ −1 (***)^	1497

(*) *A* is given by the sum of three areas: 1) the lateral area of a truncated cone having *D* _i-nozzle_ and *D* _f-nozzle_ as bases and *L* _act_ as height; 2) the lateral area of a truncated cone with *D* _tip_ and *D* _o_ as bases and *H* _tip_ as height; 3) the annulus area with *D* _tip_ and *D* _f-nozzle_ , respectively as external and internal diameter;

(**) *I* = *P* / *A* ;

(***) from Barba et al [ 8 ] .
